# Supplementary figures and images for: Staphylococcus aureus-induced immunosuppression mediated by IL-10 and IL-27 facilitates nasal colonisation
Source: PLoS Pathog. 2022 Jul 1;18(7):e1010647. doi: 10.1371/journal.ppat.1010647 (PMC9282462; doi:10.1371/journal.ppat.1010647)

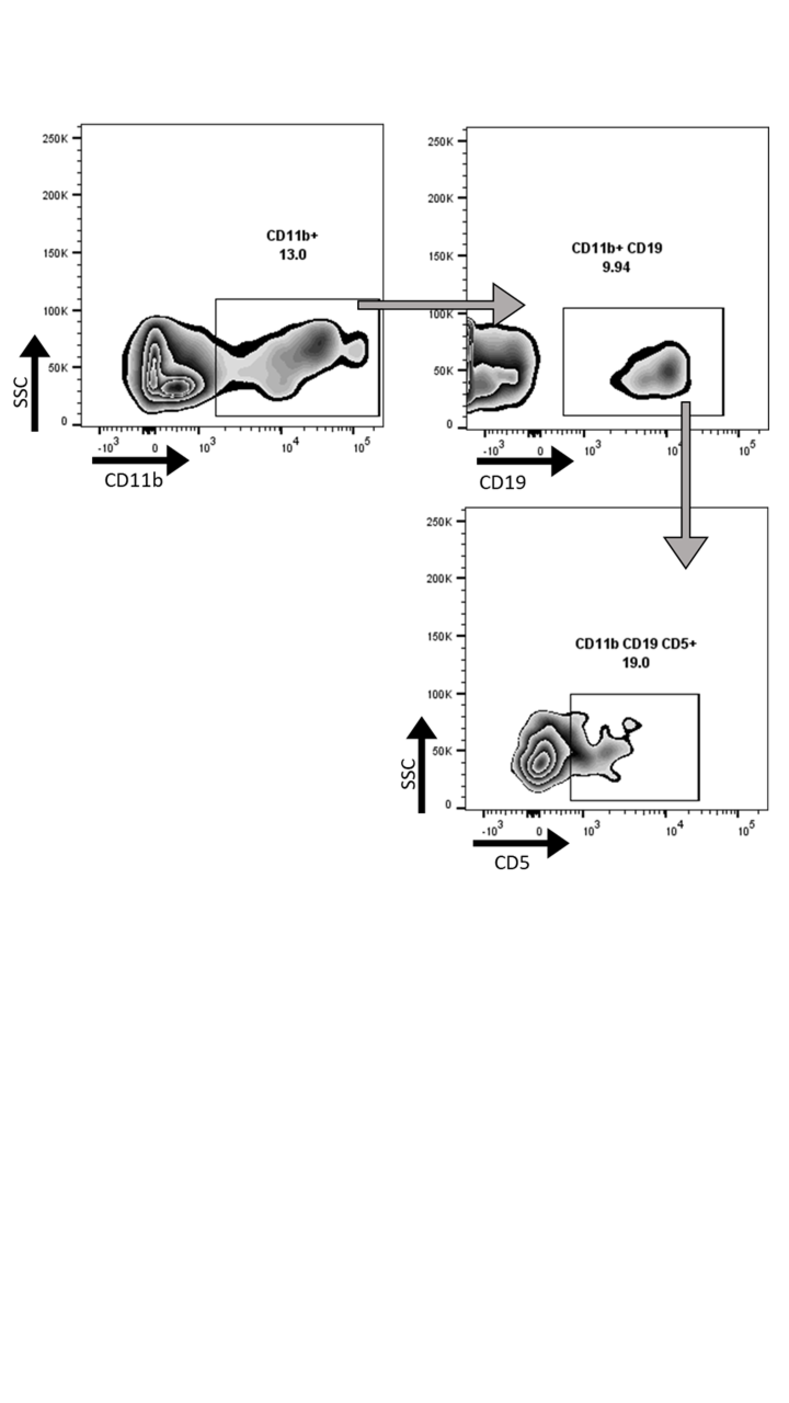

Supplement: S1 Fig — Wild-type mice were intranasally colonised with S. aureus Newman SmR (2 × 10^8^ colony-forming units/nose). At 6h mice were culled, NT tissue was excised and digested for flow cytometry analysis. Cells were gated on single, live CD45+ cells> CD11b+> CD9+> CD5+. A representative FACs plot of this population is shown (Experimental unit = 1 mouse, total # mice used 5, experiment was performed once), indicating % cell populations. (TIF) [file ppat.1010647.s001.tif]

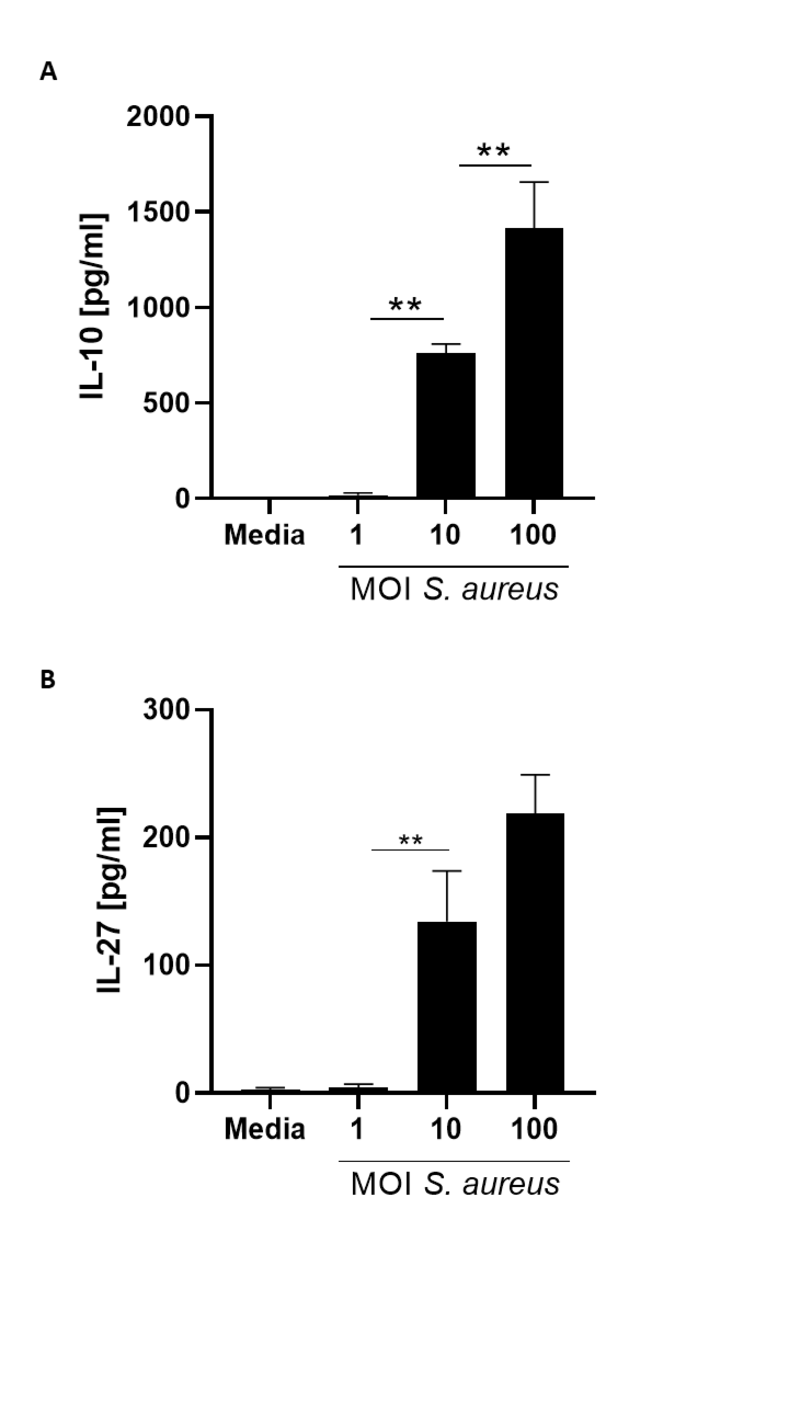

Supplement: S2 Fig — WT BMDMs were exposed to S. aureus Newman at MOI 1, 10 or 100 for 3 h. Cells were washed with gentamicin to remove extracellular bacteria. Cells were then further incubated for 24 h and levels of IL-10 (A) and IL-27 (B) were determined by ELISA. Values are expressed as mean protein concentration ± S.E.M. (Experimental unit = BMDMs isolated from 1 mouse, n = 8–10 per group, total # animals used 10, data generated from 8–10 independent experiments). Statistical analysis was carried out by one-way ANOVA, and student t-test. **P≤0.01. (TIF) [file ppat.1010647.s002.tif]

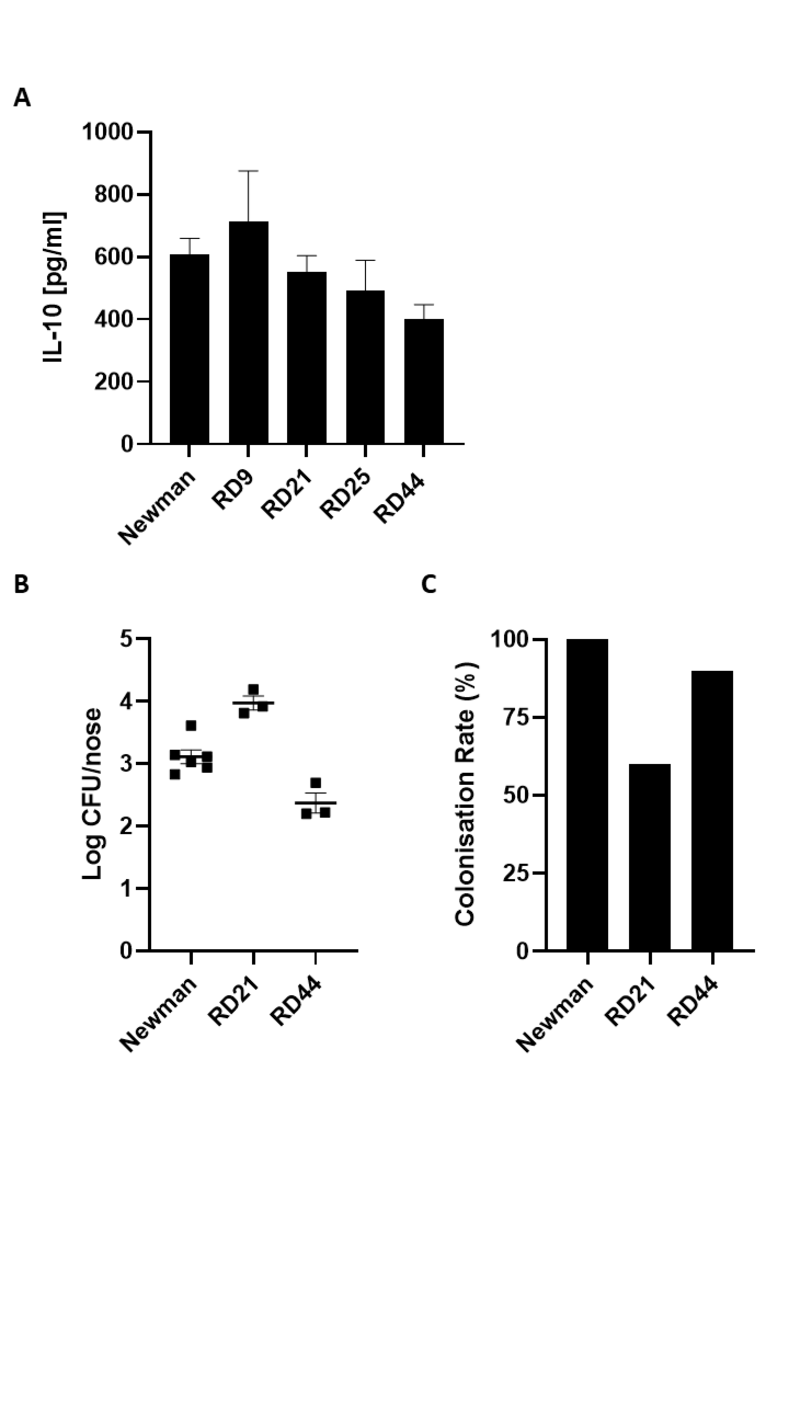

Supplement: S3 Fig — WT BMDMs were exposed to S. aureus strains RD9, RD21, RD25, RD44 at MOI 10 for 3 h. Cells were washed with gentamicin to remove extracellular bacteria. Cells were then further incubated for 24 h and levels of IL-10 were determined by ELISA (A). Values are expressed as mean protein concentration ± S.E.M. (Experimental unit = BMDMs isolated from 1 mouse, n = 4 per group, total # animals used 4, data generated from 4 independent experiment). WT mice were intranasally colonised with S. aureus Newman SmR, RD21 SmR and RD44 SmR (2 × 10^8^ colony-forming units/nose). At 10 days mice were culled, noses were homogenized and serial dilutions of homogenates were plated onto streptomycin-supplemented TSA plates. Plates were grown overnight and CFUs were enumerated. Results are expressed as Log CFU/nose (B) and colonisation rate (C) as determined by the number of mice colonised by S. aureus/total number of mice. (Experimental unit = 1 mouse n = 3–6 mice per group, total # animals used 12, data generated from 2 independent experiment). (TIF) [file ppat.1010647.s003.tif]

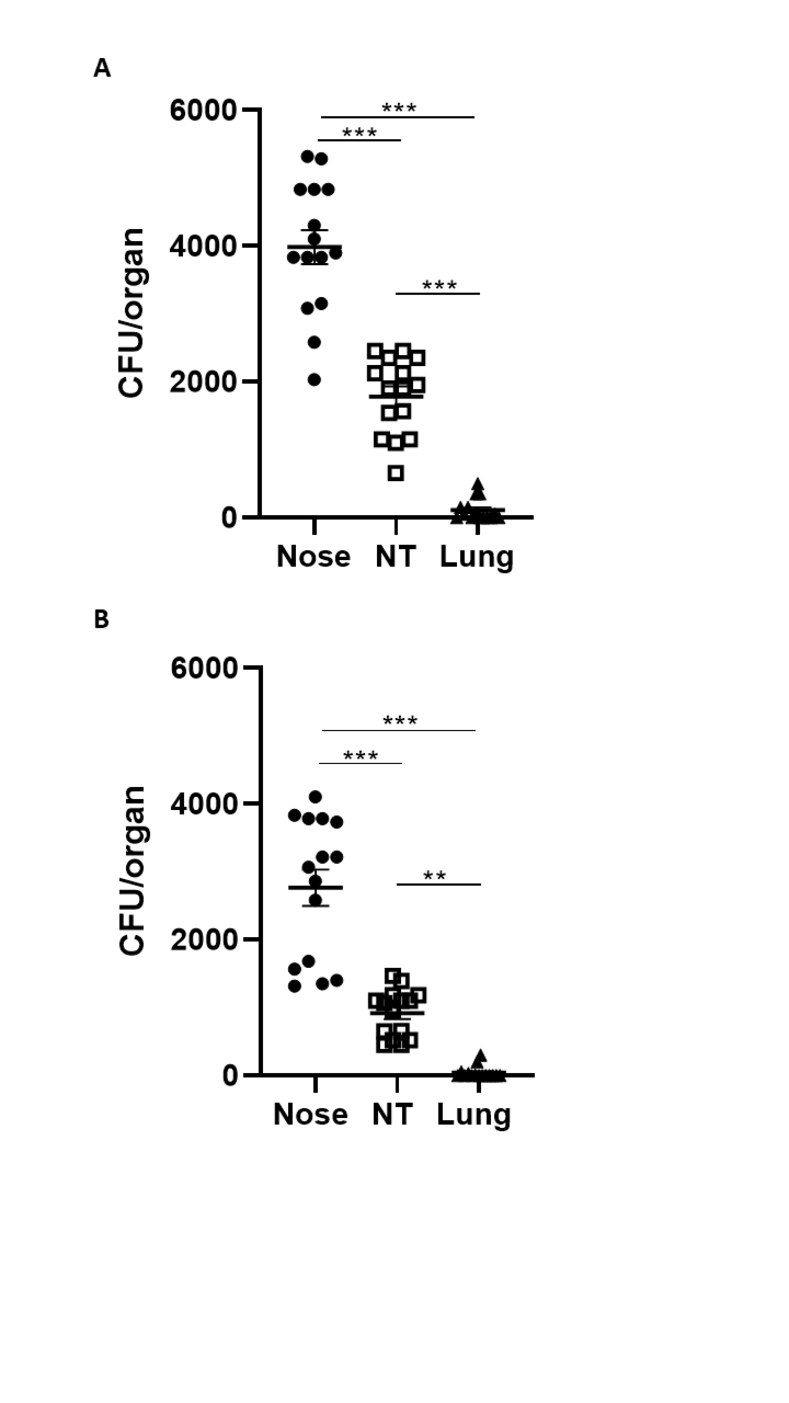

Supplement: S4 Fig — Wild-type mice were intranasally colonised with S. aureus Newman SmR (2 × 10^8^ colony-forming units/nose). At 3 days (A) and 7 days (B) mice were culled and noses, nasopharyngeal tissue and lungs were excised. Tissue was homogenized and serial dilutions of homogenates were plated onto streptomycin-supplemented TSA plates. Plates were grown overnight and CFUs were enumerated. Results are expressed as CFU/organ (Experimental unit = 1 mouse n = 15 per group, total # animals used 30, data generated from 3 separate experiments,). Statistical analysis was carried out by one-way ANOVA, and student t-test. **P≤0.01, *** P≤0.001. (TIF) [file ppat.1010647.s004.tif]

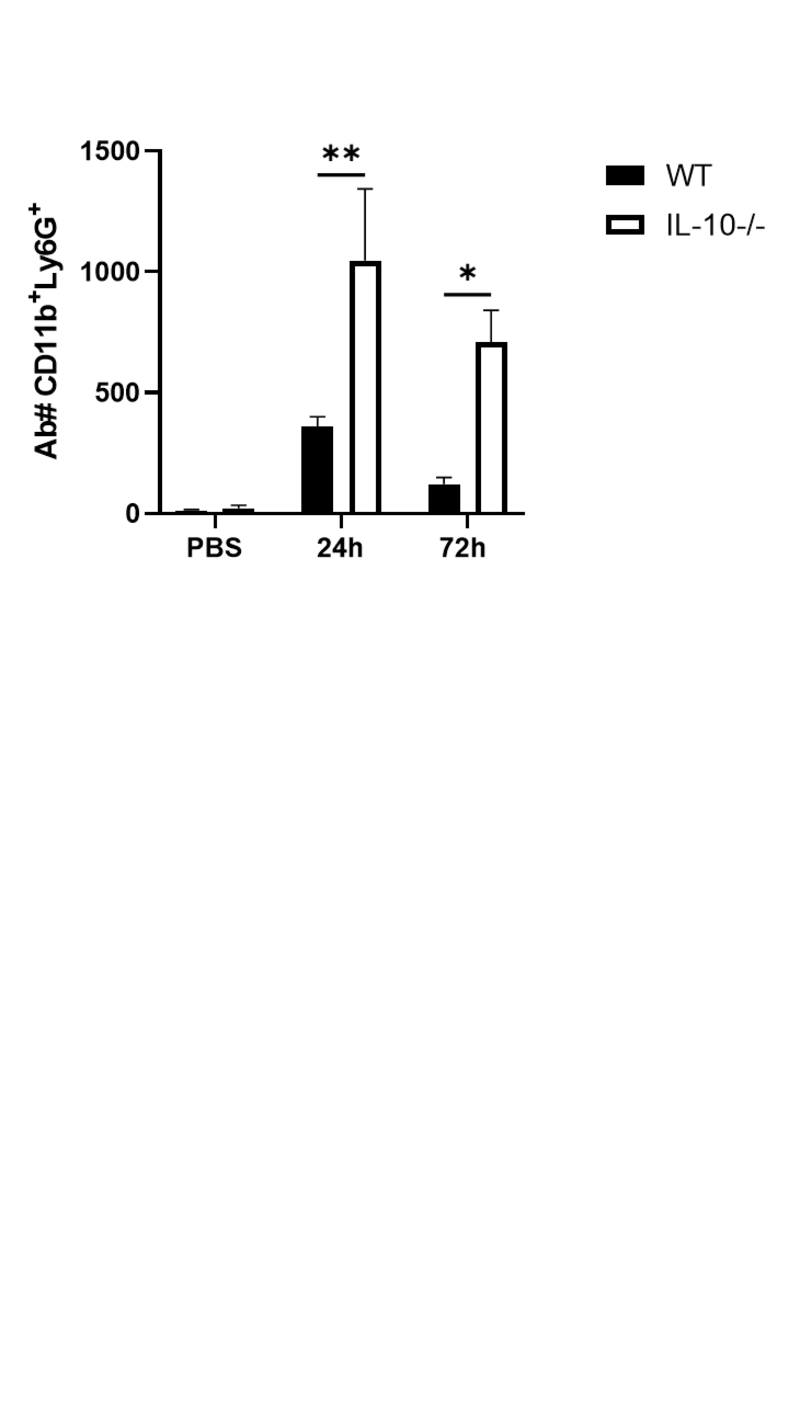

Supplement: S5 Fig — Wild-type and IL-10-/- mice were intranasally colonised with S. aureus Newman SmR (2 × 10^8^ colony-forming units/nose). At 24h and 72h mice were culled, NT tissue was excised and digested for flow cytometry analysis. Cells were gated on single, live CD45+ cells> CD11b+> Ly6G+ cells. Results are expressed in absolute cell numbers (Ab #) with mean ± S.E.M. (Experimental unit = 1 mouse, n = 4 per group, total # animals used 24, data generated from 2 independent experiments). Statistical analysis was carried out by two-way ANOVA. ** P≤0.01, *P≤0.05. (TIF) [file ppat.1010647.s005.tif]

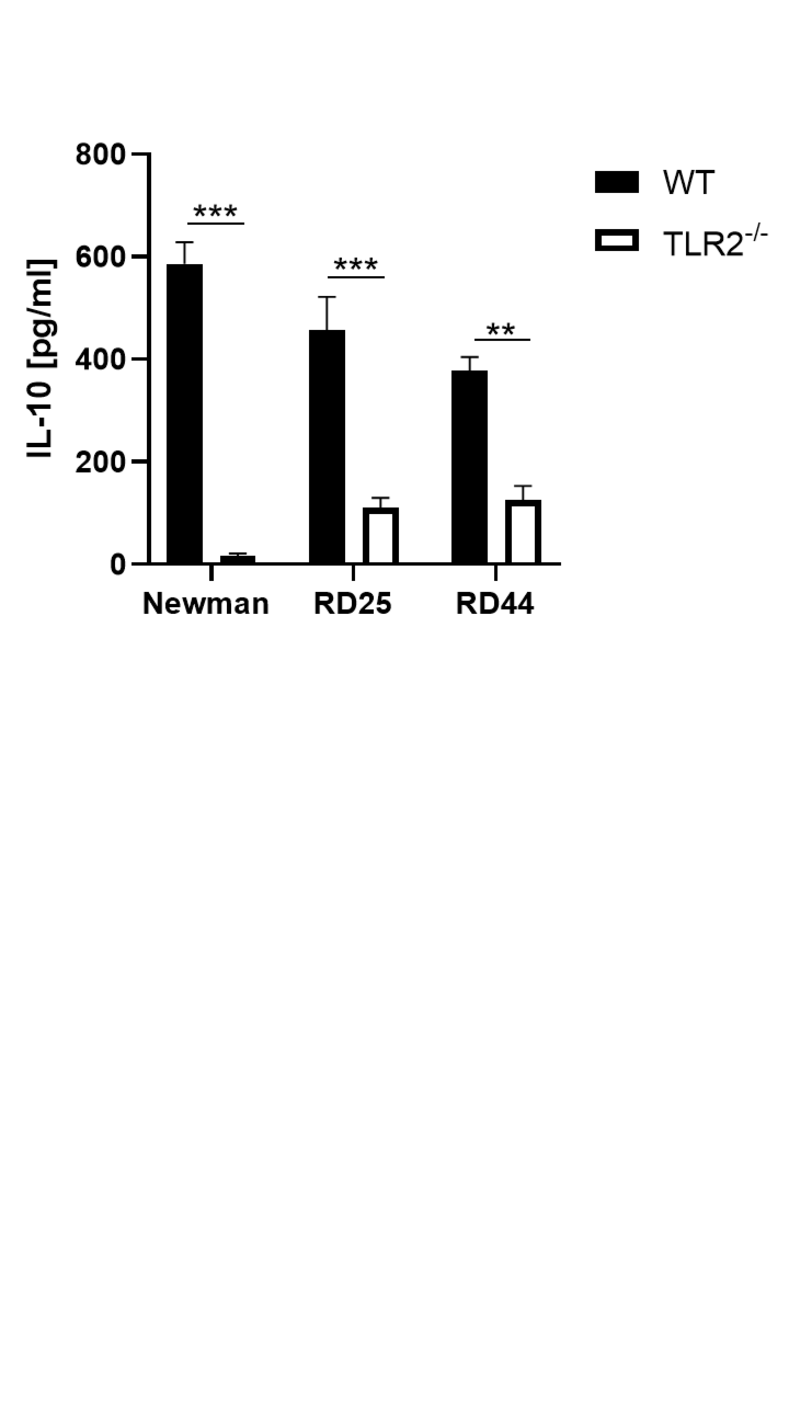

Supplement: S6 Fig — WT BMDMs or TLR2-/- BMDMs were exposure to S. aureus strains RD25, RD44 at MOI 10 for 3 h. Cells were washed with gentamicin to remove extracellular bacteria and were then further incubated for 24 h and the levels of IL-10 were determined by ELISA. Values are expressed as mean protein concentration ± S.E.M. (Experimental unit = BMDMS isolated from 1 mouse, n = 3 per group, total # animals used 6, data generated from 3 independent experiments. Statistical analysis was performed using one-way ANOVA. ** P≤0.01, *** P≤0.001. (TIF) [file ppat.1010647.s006.tif]
