# Supplementary material for: Staphylococcus aureus-induced immunosuppression mediated by IL-10 and IL-27 facilitates nasal colonisation
Source: PLoS Pathog. 2022 Jul 1;18(7):e1010647. doi: 10.1371/journal.ppat.1010647 (PMC9282462; doi:10.1371/journal.ppat.1010647)
Supplement: Attachment — Submitted filename: Kelly et al. Response to reviewers.docx [file ppat.1010647.s007.docx]

Response to Reviewers

**Reviewer 1**

***Overall, the manuscript addresses a relevant question in the S. aureus field, providing a significant advance in our understanding of the interplay between the bacterium and the host immune system. The experiments are for the most part appropriately designed, and include relevant controls . In most instances, the conclusions are supported by the data. There are a few concerns related to strain choice and data interpretation that should be addressed by the authors.***

We thank the reviewers for acknowledging the strengths of this study and their helpful suggestions as to how the manuscript could be improved which we have addressed below.

***1. The authors conduct all studies with strain Newman. In principle this is fine, however it would be informative to know if the observed phenotypes occur in a non-Newman strain. At minimum, the authors should discuss this limitation to the study.***

Strain Newman was chosen for our studies due to the fact that this is the original strain used in the establishment of the model of *S. aureus* murine nasal colonisation (1) and we have previously utilised this strain to interrogate host immune responses underpinning nasal colonisation (2-3). We now include a new supplemental figure (S3 Fig) which demonstrates the production of IL-10 by BMDMs in response to a panel of *S. aureus* human colonising isolates. These results indicate that the majority of colonising strains have the capacity to drive a robust IL-10 response that is comparable to the experimental strain Newman. Furthermore we demonstrated that 2 of these colonising strains were capable of colonising mice at a similar level to the Newman strain. Discussion of this point has been added at appropriate points in the manuscript.

***2. The evidence in support of upregulation of il10 transcript in B cells, macrophages, and MDSC (Fig 1F) is reasonably compelling (although no stats were provided – see below). However, the same data in Figure 4F for il27 is not so strong and hovers right at the cut-off level. The authors comment confidently that the sources of IL-27 are these cell types (and is part of the basis for their autocrine regulation hypothesis), but it is not clear the data fully support this conclusion. Could there be another CD45+ cell type in vivo that is producing IL27? The data from the figure with purified BMDM supports the autocrine model, but this may not be what happens in vivo? The authors may consider adjusting their interpretations or discussing this alternative possibility in light of the strength of the data.***

The data presented in figure 4F suggests that IL-27 is being produced by multiple myeloid cells within the nasal cavity. Our in vitro data strongly support the notion that IL-27 acts in an autocrine manner to control IL-10 production by macrophages. It is likely that it acts in a similar manner to control IL-10 production by B-cells and MDSC. It is possible that there are other leukocyte subsets present within the nasal cavity that contribute to IL-27 production and this point has now been highlighted in the revised manuscript.

***3. Lines 228 to 230. The simplest and most accurate interpretation of the data is that the IL-27 signal peaks prior to IL-10. One possibility, of many, is that IL-27 could act upstream to impact levels of IL-10. The authors will test this possibility in the following section, but they should consider only stating what can be firmly concluded from the results in Figures 1 and 4 until they have data to support this specific possibility.***

We accept this point and apologise for over interpreting the data at this point in the paper. We removed the relevant section of text.

***4. It is surprising that no time points earlier than three days were examined in the nasal colonization comparisons between WT and IL10-/- mice. Experiments in figure 1 look as early as six hours and begin to show differences in IL-10 and IL-27. What does colonization look like at very early timepoints, maybe 6 or 12 hours? This information would also be helpful to establish whether or not S. aureus ever initially colonizes an IL10-/- mouse (by day 3 there are very few bacteria remaining).***

We now include a revised Figure 2 which demonstrates that WT and IL-10-/- mice are initially colonised to the same extent. The more rapid clearance of *S. aureus* from the nasal tissue in IL-10-/- mice only becomes apparent by day 3 post colonisation consistent with the peak of the IL-10 responses occurring at 24 hours.

***Minor comments***

***1.Line 61: Consider changing microflora to microbiota.***
Text modified.

***2. Lines 171-172: The authors should stick with one convention for colonised/colonized. It is used interchangeably in places.***
Test modified throughout.

***3. Line 321. UMAS-1 should be UAMS-1.***
Text modified.

***4. Were statistics done on Figs 1E-F and Fig 4E-F? Some reasonably strong conclusions are made about these data. If statistical significance was not achieved, the authors should appropriately consider toning things down and pointing out that statistical significance was not achieved.***
Statistical analysis was performed on the data in Fig 1E and 4E which demonstrates a statistically significant increase in IL-10 and IL-27 expression between immune cells isolated from the NALT vs. the nose and also between CD45+ vs. CD45- cells within the NALT. We have clarified this point in the relevant section. We apologise that this was inadvertently omitted in the original summitted draft. We did not perform any statistical analysis on the data in figures 1F and 4F since the point here was not to draw any comparison between the levels of cytokines produced by individual cell subsets, rather to establish what cells might be contributing.

***5. Please define the abbreviation Ab# in the figure legend of figure 3.***

Legend modified.

**Response to Reviewer 2**

***Overall this is a well-constructed study with data supporting a role for S. aureus-mediated induction of IL-10 via IL-27 in slowing the rate of S. aureus nasal clearance in a mouse model of nasal colonization. The experiment blocking IL-27 in vivo in WT vs IL-10-/- mice with intranasal administration of anti-IL-27 antibody compared with isotype provides particularly strong support that IL-27 induces the IL-10 response to S. aureus Newman in the nose. (It also suggests that perhaps the key events are occurring in the mucus layer/lumen.)***

We thank the reviewer for their complimentary comments on our manuscript.

***The focus entirely on a single, domesticated strain of S. aureus is a downside to a well-constructed set of experiments that support a role of S. aureus-mediated induction of IL-10 in preventing more rapid nasal clearance in a mouse nasal colonization model. Use of a primary nostril isolate, or clinical isolate, rather than Newman, or, at the least, validation of a key finding with a primary nasal isolate in at least one experiment would strengthen the manuscript and broaden the implications of the findings.***

Please see response to reviewer 1 above (point 1). We now include a new data (S3 Fig) that demonstrates robust IL-10 induction by a panel of *S. aureus* strains isolated from persistent nasal carriers.

**Minor issues**

***A major question that could be at least raised in the Discussion is whether this host immunological response is specific to S. aureus nasal colonization or does it occur with other more common human-associated nasal bacteria, e.g., Staphylococcus epidermidis or nasal-associated non-diphtheriae Corynebacterium species. Similarly, do other common nasal colonizers, many of which have higher prevalence in the human population than S. aureus, alter the host IL-27 and IL-10 response? In the long run it seems key to determine whether the possibility of altering the host response by dampening IL-10 to increase S. aureus clearance would also impact more benign nasal colonizers in an undesirable manner***

We agree with the reviewer that it is possible that the induction of immunoregulatory cytokines such as IL-10 and IL-27 is a common mechanism shared by commensal species to promote persistent colonisation and we have expanded the discussion of this in the introduction. Exploring these responses in the context of other respiratory tract commensal bacterial species is outside the scope of the current study.

***For Fig. 1, why were nose and nasopharyngeal tissue analyzed separately? Please add this information to the Results section. Also, please indicate in the Methods, lines 441-444, how, and using what anatomic landmarks, the nose and nasopharyngeal tissue were separated from each other for analysis.***

*S. aureus* has been shown to colonise both the outer most anterior nares (nose) and the deeper nasopharyngeal tissues, and this point has been highlighted in the revised manuscript. Therefore it was important to study host immune responses at both site. We provided further detail in the methods section as to how both tissues were isolated in the methods section.

***Since S. aureus colonization burden is highest in the nose in this mouse model (Fig S1), it is unclear why the analysis for IL-22+ and IL-17+ T cells (Fig. 3) was done using nasopharyngeal tissue rather than nose tissue.***

Our data is consistent with previous studies which show that *S. aureus* has the capacity to colonise both the nose and nasopharyngeal tissue. These two anatomical sites are in close proximity, however the NT consists of mainly CD45+ immune cells while the nose has a lower number of immune cells present. Given the limited tissue that can be recovered from a mouse’s nose it is very difficult to isolate sufficient numbers of leukocytes from this tissue to perform meaningful flow cytometry analysis. Consistent with previously publised studies that have examined immune responses within the nasal cavity during *S. aureus* colonisation, we performed our detailed analysis of T-cell responses using leukocytes isolated from the nasopharyngeal tissue where both IL-22 and IL-17 have been shown to be expressed (3-5).

***In comparing Figure 4 (IL-27) and Figure 1 (IL-10), for which the same animals were used, what might be inducing an increase in IL-10 in the nose (Fig 1D) in S. aureus colonized animals in the absence of a detectable induction of IL-27 (Fig 4D)?.***

Our study identifies an important role for TLR2 signalling in mediating *S. aureus* driven IL-10 production by macrophages, which is consistent with previously published studies. We propose that during *S. aureus* colonisation *S. aureus* also induces IL-27 which serves to amplify or promote this IL-10 response. In the absence of TLR2, IL-27 is unable to enhance IL-10 production (Fig 6C) however there is evidence that during *S. aureus* nasal colonisation in vivo, blockade of IL-27 does not return the local IL-10 response to baseline (Fig 5A). It is likely that this residual non IL-27 driven response is as a direct result of *S. aureus* PAMPS engaging TLR2. We apologise that this message was not clear in our original manuscript and have attempted to clarify it in our revised version.

***Abstract & Line 62: 30% persistent S. aureus colonization picks the high side of what exists in different studies and is very likely an overestimate. A range or a mid-point would be more appropriate.***

The text has been modified accordingly.

***Is the mechanism responsible for the almost 5 log drop in S. aureus from the 2x10^8 inoculum to ~4x20^3 level on Day 3 (Fig. 2) known?***

A limitaiton of this model is that a very high inoculum must be utilised to obtain stable levels of colonisaiton and that colonisation is transient. This is consistent with what has been shown in previous publications that have utilised this model. The model relies on the mouse inhaling the bacteria into their nostrils while sedated and the efficiency of this process is such that the animals are rarely colonised with the total 2x10^8^ CFU. We now include a new Fig 2 which demonstrates that both WT and IL-10-/- mice are initially colonised with *S. aureus* to similar extents at a level of ~6 Log at 3 hours post challenge. Colonisation begins to steadily decline between 24-72 hours presumably as the host immune response kicks in at a sufficient rate to facilitate clearance of the bacteria. IL-17 responses for example, which have been shown to be critical for controlling bacteria clearance in this model (4,5) are elevated on day 3 (Fig 3) and have previously been shown to be elevated in the tissue as early as day 2 and (4). In the absense of IL-10 this immune response is activated to a greater extend resulting in a more rapid clearance of the bacteria.

***Please clarify in the abstract that data are based on the use of a mouse model of S. aureus nasal colonization.***

Text modified.

***Please clarify the # of animals needed per group which is listed in Line 423 as 5 animals/group/experiment x2-3 experiments, which is 10 or 15 per group, but in Line 456 as ~15 animals/group.***

Text modified.

***Please include an indication of how many independent experiments were done for panels A-D in Figures 1 & 4 as well as for Figure 2, Figure 3, Figure 5, and Fig S2.***

Figure Legends modified.

***Any thoughts as to why the IL-10 protein levels at 24 hours in Figure 5 are greater than 4 times lower than those in Figure 1?***

This is most likely due to experimental variability. The experiments presented in Fig 5 and Fig 1 were performed at different stages in the project. Importantly the relevant controls are included in each experiment which allow us to draw robust conclusions on the role of IL-10 from the data, despite the levels quantified in the tissue varying between experiments. ***Is there a typo on line 804 regarding the total animals used?***

Apologies for this oversight. The text has been edited accordingly.

***Consider using microbiota rather than microflora, as the former is technically more accurate.***
Text modified.

***Line 165: define the abbreviation MDSC when first used.***

Text Modified.

**Response to Reviewer 3**

***However, in my opinion this manuscripts draws a comprehensive picture of the inflammatory surrounding created within the nasal cavity upon colonisation of S. aureus. The host-immunity associated with nasal colonisation is in general neglected and this study represents in important extension to our current understanding.***

We thank the reviewer for identifying this as an important study that draws comprehensive conclusions.

***However, I have two major concerns regarding the experimental procedures. It is well appreciated that S. aureus strains possess strikingly different abilities to cause inflammation, this depends on the activity of various cellular machineries. The authors conducted their experiments using the single strain S. aureus Newman and verified in their experiments, that TLR 2-signalling is important for IL-10 production. TLR2 stimulation differs between lineages. For example lipoprotein shedding is shaped by agr-activity and their degradation by lipases can dampen TLR2 activation (Chen and Alonzo 2019). In strain Newman a phospholipase is inactivated by phage integration (Bea…Schneewind, 2006), entailing increased TLR2 activation. The authors should verify (at least in vitro) using a panel of S. aureus strains that the effects of S. aureus on IL-10 production are widely conserved between strains.***

We now include a new data (new S3 Fig) that demonstrates robust IL-10 induction by a panel of *S. aureus* strains isolated from persistent nasal carriers. Please see response to reviewer 1 above (point 1). In addition, we validated that IL-10 production in response to colonising strains is also TLR2 dependent. The reduction in IL-10 production by TLR2-/- BMDMs in response to the human colonising strains was similar to that seen with *S. aureus* strain Newman (new S6 Fig).

***Secondly, the authors should attempt to verify their experiments in humans. It might be possible to determine IL-10 levels in the nasal cavities of humans colonized with S. aureus vs humans lacking S. aureus.***

We agree with the reviewer that this would be an excellent next step, however it is significantly beyond the scope of the current study. In support of our work however it has previously been shown that PBMCs isolated from persistent carriers exhibit a lower IFN-g to IL-10 mRNA ratio when exposed to *S. aureus* in vitro (6), while PBMC from persistent carriers produced increased levels of IL-19, an IL-10 cytokine family member, upon exposure to endogenous colonising isolates vs non endogenous strains (7). In addition other regulatory cytokines such as IL-35 have been shown to inhibit effector Th17 responses in human nasopharynx-associated lymphoid tissue (8). These studies strongly support the notion that *S. aureus* nasal carriage in humans is also associated with the induction of tolerogenic responses. We have expanded our discussion on this point in the revised manuscript. Additionally we now include new data (S3 Fig) which demonstrates the ability of a panel of *S. aureus* strains isolated from persistent nasal carriers to induce robust IL-10 responses in macrophages.

**Minor Comments:**

***Fig. E the colour of the bars and the legend seems not to match.***

We apologise for this oversight, the figure has now been amended.

***Fig4 F: The expression levels in the B-cells and myeloid cells appears not to differ significantly from the baseline. Does this result really allow the interpretation that these cells produce the IL upon S. aureus colonisation?***

Identifying the precise cellular source of IL-27 in vivo is a challenge, the data presented in Fig 4F serve to prove the point that multiple cell lineages are likely contributing to its production in vivo. Our in vitro data strongly support the notion that IL-27 acts in an autocrine manner to control IL-10 production by macrophages. It is likely that it acts in a similar manner to control IL-10 production by B-cells and MDSC. It is possible however that there are other leukocyte subsets present within the nasal cavity that contribute to IL-27 production and this p. We have tempered the conclusions drawn from the data presented in Fig 4F.

***The result shown in FigS3 is describe discussion the first time. It should be moved to the results part.***

We have made this change.

**Response to Editor Comments**

***All three reviewers requested the use of primary nasal or clinical S. aureus isolates in addition to the lab strain Newman and they asked for some additional control experiment. Moreover, they suggested modifications concerning the presentation and discussion of your data.***

We now include new data involving primary nasal isolates from *S. aureus* persistent carriers and have made the relevant additions/modifications requested by all 3 reviewers.

***In addition to the reviewers’ comments we ask you to discuss potential mechanisms leading to IL-10 and IL-27-dependent loss of S. aureus from nares more thoroughly. You currently suggest that neutrophils, AMPs, and altered expression of epithelial attachment sites could impair S. aureus nasal colonization. However, there are probably not enough neutrophils in the nasal cavity to eradicate S. aureus directly and the nasal IgA cannot facilitate pathogen detection and phagocytosis. Moreover, AMPs are more or less equally active towards S. aureus and commensals and can hardly explain a selective inhibition of S. aureus. In the and, neutrophil and T-cell mediated mucosal inflammation may alter the abundance attachment sites and of nutrients on the nasal epithelium in a way that is unfavourable for sustained S. aureus colonization.***

A previously published study has clearly demonstrated that IL-17 driven neutrophil recruitment to the nasal tissue is required to control nasal colonisation by *S. aureus* in this model (4). In that study the authors demonstrate that neutrophil migration into the nasal cavity was reduced in IL-17-/- mice and that following neutrophil depletion colonisation rates were significantly increased. In a follow up study the same authors demonstrate that expression of antimicrobial peptides in the nasal tissue were also reduced in IL-17-/- mice (5). Our own previous studies identified an important role for IL-22 in controlling *S. aureus* nasal colonisation though this cytokines ability to directly regulate local antimicrobial peptide expression in the nasal cavity but also to modulate expression of loricrin and important ligand for *S. aureus* adherence to this tissue (3). In that study we demonstrate a significant reduction in AMP expression but not neutrophil infiltration in IL-22-/- mice as compared to WT mice that is associated with an increased bacterial burden. Thus the published literature suggest that there are multiple host immune defence pathways that are activated in response to *S. aureus* exposure within the nasal cavity which function in concert to control *S. aureus* persistence at this site. Given the important and well documented role for IL-17 and IL-22 in controlling *S. aureus* nasal colonisation we set out to establish how both of these pathways might be counter regulated by IL-10. In the absence of IL-10 we observed enhanced IL-17 and IL-22 responses that are associated with a reduction in bacterial burden within the nasal tissue likely through these cytokines combined proven effects on neutrophil infiltration, AMP expression and modulation the nasal epithelium structure. We have modified the text in a number of place throughout the manuscript to better convey this message. In addition we include new data that demonstrates reduced numbers of neutrophils present within the nasal tissue in WT mice compared IL-10^-/-^ mice on day 3 post *S. aureus* colonisation (S5 Fig).

***We have problems though to think of a direct elimination process. Terms such as ‘clearance’ (at several positions in the manuscript) or ‘eradication’ (lines 84, 379) may not be appropriate as long as there is no evidence for direct elimination.***

We have modified the text throughout the manuscript to temper our conclusions regarding elimination of the bacteria. However we and others have previously reported effective clearance of *S. aureus* from the nasal cavity in this model after 21-28 days (3,4).

**References cited.**

1. Kiser *et al.* Development and characterization of a Staphylococcus aureus nasal colonization model in mice. Infect Immun 1999 Oct;67(10):5001-6.

# 2. Mulcahy *et al.* Nasal colonisation by Staphylococcus aureus depends upon clumping factor B binding to the squamous epithelial cell envelope protein loricrin. PLoS Pathog. 2012 Dec;8(12):e1003092.

3. Mulcahy ME, *et al.* Interleukin-22 regulates antimicrobial peptide expression and keratinocyte differentiation to control Staphylococcus aureus colonization of the nasal mucosa. Mucosal Immunol. 2016;9(6):1429-41.
4. Archer NK, *et al.* Clearance of Staphylococcus aureus nasal carriage is T cell dependent and mediated through interleukin-17A expression and neutrophil influx. Infect Immun. 2013;81(6):2070-5.
5. Archer NK, *et al*. Interleukin-17A (IL-17A) and IL-17F Are Critical for Antimicrobial Peptide Production and Clearance of Staphylococcus aureus Nasal Colonization. Infect Immun. 2016;84(12):3575-83.
6. Nurjadi D, *et al.* Ratio of T-Helper Type 1 (Th1) to Th17 Cytokines in Whole Blood Is Associated With Human beta-Defensin 3 Expression in Skin and Persistent Staphylococcus aureus Nasal Carriage. J Infect Dis. 2016;214(11):1744-51.
7. Reiss-Mandel A, *et al.* Staphylococcus aureus Colonization Induces Strain-Specific Suppression of Interleukin-17. Infect Immun. 2018 Mar; 86(3): e00834-17.
8. Xu R, *et al*. IL-35 is critical in suppressing superantigenic Staphylococcus aureus-driven inflammatory Th17 responses in human nasopharynx-associated lymphoid tissue. Mucosal Immunol. 2020;13(3):460-70.
